# Anaesthesia for the separation of conjoined twins

**DOI:** 10.4103/0019-5049.79902

**Published:** 2011

**Authors:** Jaya Lalwani, KP Dubey, Pratibha Shah

**Affiliations:** Department of Anaesthesiology and Critical Care, PT.J.N.M. Medical College and Dr. Bram Hospital, Raipur, Chhattisgarh, India

**Keywords:** Anaesthesia, conjoint twins, thoraco-omphalopagus

## Abstract

Thoraco-omphalopagus is one of the most common type of conjoint twins accounting for 74% cases of conjoint twins. We report the anaesthetic management for successful separation of thoraco-omphalopagus conjoint twins, both of them surviving till date. We highlight the responsibility of anaesthesia team in anaesthetising the two individual patients simultaneously, need of careful monitoring and anticipation of complications like massive blood loss, hypotension, hypokalemia, hypoxia and hypercabia. Detailed description of successful management is reported.

## INTRODUCTION

Conjoined twins, one of the most fascinating human malformations, have been worshipped as gods and feared as monsters. They are said to result from an aberrant twinning process with incomplete fission of the zygote’s primitive streak at 20 days ovulation.[1,2] Their incidences are very rare (1:200,000) live births.[3] Classification of conjoined twins is based on the site of union as thorax–40% (thoracopagus), upper abdomen–(xiphopagus), lower abdomen–33% (omphalopagus), sacrum-19% (pyopagus), pelvis-6% (ischiopagus) or skull-2% (craniopagus).[4] Thoraco-omphalopagus is one of the most common types accounting for 74% cases. Inspite of their rare occurrence several successful operative separations have been reported.[5–11] Twinning is more common in Indian and African populations than in Caucasians.[11] We report the anaesthetic management of successful separation of thoraco-omphalopagus conjoined twins with both of them surviving till date.

## CASE REPORT

6 days old male twins weighing 3 kgs were admitted to our hospital and they were posted for surgery at 10 month of age when they were weighing 17 kgs.

**Figure 1:**
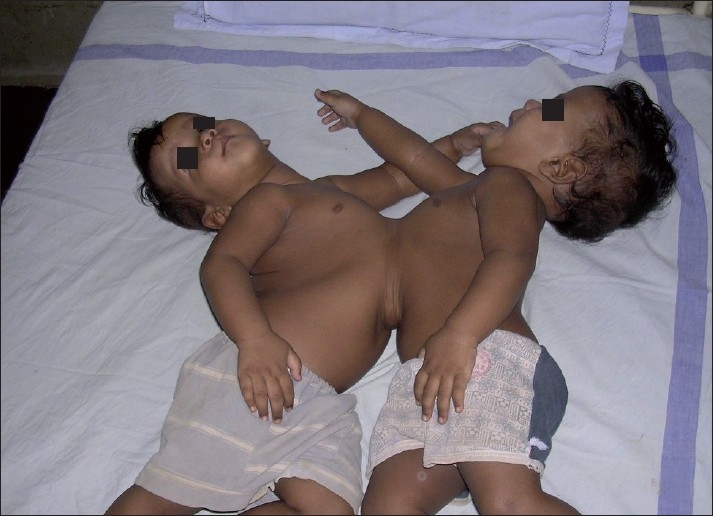
Twins at 9 months

**Figure 2:**
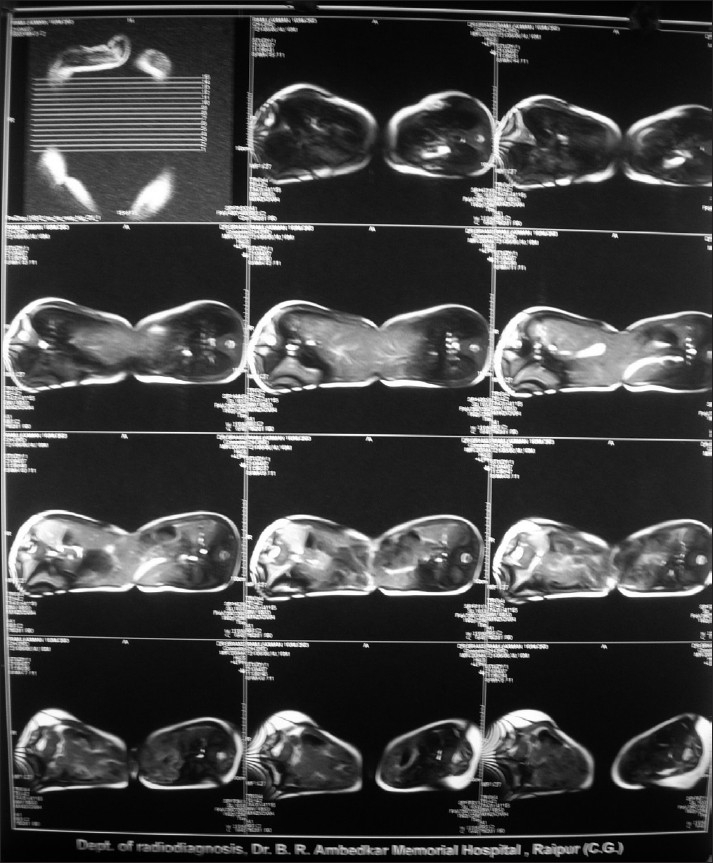
Computerise Tomography scan

They were thoraco-omphalopagus twins, joined ventrally from the manubrium sterni to umbilicus [Figure 1]. For evaluation of the extent of shared organ systems the twins were anaesthetised twice before undergoing surgery, once for undergoing spiral computerized tomography (CT) scan with contrast at 15 days and magnetic resonance imaging (MRI) at 45 days of age. The twins were named R1 and R2 to avoid confusion. At each occasion separate intravenous (i.v.) lines were secured and i.v atropine (0.15 mg) and i.v midazolam was given for premedication to R1. No appreciable change was noticed in the level of consciousness in R2 suggesting little cross-circulation. R2 was also premedicated and i.v ketamine was given to both with oxygen supplementation by mask. CT scan revealed two separate livers in each baby with fused anterior surface [Figure 2]. Separate major hepatic veins were present in each liver with no major anastomosing vessels across the liver. Hearts, spleens, kidneys appeared independent and well functioning in both. MRI and ultasonography findings confirmed the findings of CT scan.

Barium meals showed a separate gastrointestinal tract for each twin. The twins revealed asynchronous pulses and two ventricular complexes on electrocardiography (ECG), indicating that each twin had a separate individual heart. Hormonal assay suggested independent and normal function in the thyroid and adrenal cortex. Haematological and biochemical tests were within normal limits.

The preoperative investigations showed that the twins had a conjoined liver with separate biliary systems.

**Figure 3:**
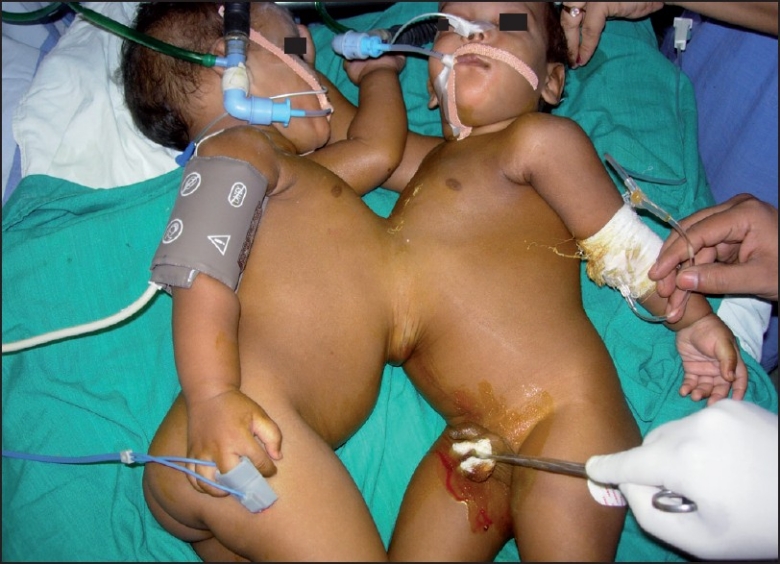
Twins after intubation

A full rehearsal of the parts to be played by each member of the anaesthesia team was carried out on the day before operation. The twins were placed on warm gamgee on the operation theatre (OT) table. ECG, non-invasive blood pressure, pulse oximeter, temperature, end tidal CO_2_ andurine output monitoring were attached separately to both the babies. In order to prevent confusion, all the monitoring cables and i.v. lines were colour-coded (red for R1and blue for R2). Separate peripheral i.v. lines were secured in OT and then both the babies were premedicated simultaneously with i.v. atropine 0.15, fentanyl 10 μg and midazolam 0.5 mg. After preoxygenation with Jackson Rees modification of the Ayre’s T-piece, the babies were induced separately with inj. thiopentone sodium and paralysis achieved with suxamethonium. Orotracheal intubation with no.4.0 mm plain orotracheal tube of both babies was done. Intubation was not difficult, although the position was not an ideal one because of the junction at the chest. After fixation of the tubes each twin was given 4 mg of atracurium besylate intravenously and their lungs ventilated manually with 50% oxygen and 50% nitrous oxide and low concentration (0.5%) of isoflurane [Figure 3]. Central venous cannulation was done through the right femoral vein via Seldinger technique in both babies. It provided a secure i.v. line for both intraoperative and postoperative periods besides central venous pressure (CVP) measurements.

**Figure 4:**
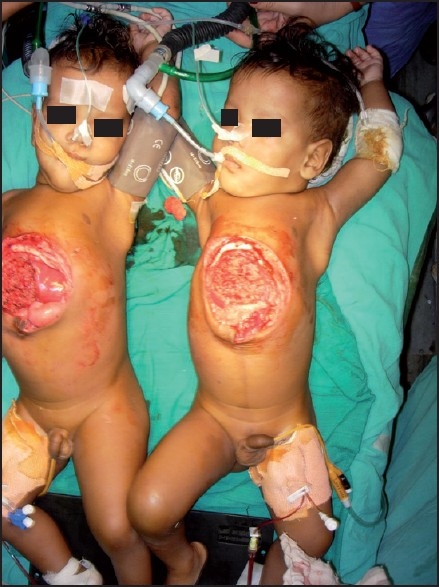
Twins after separation

Incision was made on the skin bridge connecting both twins. Surgical steps included reduction of intestinal herniation, liver resection using harmonic scalpel and separation of diaphragms. Finally remainder of connecting bridge of abdominal wall was divided thus separating the twins [Figure 4]. Blood loss was very difficult to estimate but was done by weighing the swabs, measuring the loss into the vaccum suction bottle and by serial haematocrits.

Total estimated blood loss was approximately 500 ml, which was replaced by 300 ml of fresh warm blood to each twin. After complete separation of twins, twin R2 was taken to a second operating table. At the end of the surgery residual effect of muscle relaxant was reversed with inj. glycopyrolate 0.01 mg/kg and inj. neostigmine 0.05 mg/kg to each baby. Both babies cried immediately after extubation and postoperative ventilatory support was not required. They were sent to the intensive care unit for further observation. Monitoring for haemorrhage, hypotension, hypothermia, hypocalcaemia, hypokalemia, hypoxia, hypercarbia, acidosis and CVP was continued in the postoperative period. Postoperative analgesia was done by diclonac suppositories 12.5 mg t.d.s. to both babies.

## DISCUSSION

We have described the successful separation of thoracoomphalopagus conjoint twins. The twins underwent spiral CT scan and MRI both under anaesthesia to detect organ sharing and any systemic malformations. It was advantageous as it demonstrated the extent of cross-circulation, which was not significant. As i.v. glycopyrolate and i.v. ketamine injection to R1 hardly produced any effect on R2. CT and MRI scans also confirmed our findings. It was also observed that each twin responded independently to surgical stimulus. So it was apparent that each twin could be treated as a separate entity. So we proceeded with sequential i.v. inductions, which avoided the need to ventilate one twin while trying to perform laryngoscopy on the other. We calculated drug doses based on the weight of each individual twin rather than on the combined weight.

The condition of twins in our case was stable and early separation was not indicated. So surgery was delayed till 10 months when primary immunization was complete. Rapid expansion of the body wall allowed easy closure.

The main problems during the operation were the unusual position of the patients, profuse blood loss, prolonged operation on two patients on the same table and the number of medical personnel involved. All the problems were anticipated beforehand and subsequently dealt with successfully.

The need for careful monitoring has been emphasized.[12] Frequent estimation of blood gases and CVP monitoring should be done. In the case presented intra-arterial monitoring was not used but could have been advantageous. Depending on the site of union, the position of twins at the time of tracheal intubation can be difficult. In our case due to the junction at lower chest and upper abdomen the twins could be placed in lateral position. Intubation though awkward was not difficult. Twisting heads of both babies led to a reasonably normal intubating position.

Massive blood loss should always be anticipated and prompt replacement considered. Use of harmonic scalpel greatly minimized blood loss during surgery. For blood to be transfused the objective was maintenance of each twin’s haematocrit around 40%. Maintenance of normal body temperatures in these infants during prolonged surgery is also a great challenge. Our measures included the use of warm blankets, warmed i.v. fluids and blood products, warming lights and control of the ambient temperature in the operating room. Since the hormonal assay was normal suggesting independent and normal function of the adrenal cortex steroid therapy was not given.[13] Finally adequate personnel and planning on the part of anaesthesiology team proved very helpful. Our allotment of resident and senior anaesthesiologists to each infant allowed for coordination of complex intraoperative tasks and vigilant monitoring.

After an uneventful postoperative period of 3 weeks, the children were discharged.
